# A qualitative exploration of declining sexual intimacy among married men and women

**DOI:** 10.34172/hpp.025.44424

**Published:** 2025-11-04

**Authors:** Somayeh Azimi, Mina Hashemiparast, Mahasti Alizadeh Mizani, Zeinab Javadivala, Behnam Bagherzadeh, Hamid Allahverdipour

**Affiliations:** ^1^Social Determinants of Health Research Center, Yasuj University of Medical Sciences, Yasuj, Iran; ^2^Department of Health Education and Promotion, Zanjan University of Medical Sciences, Zanjan, Iran; ^3^Social Determinants of Health Research Center, Health Management and Safety Promotion Research Institute, Tabriz University of Medical Sciences, Tabriz, Iran; ^4^Department of Health Education & Promotion, Tabriz University of Medical Sciences, Tabriz, Iran; ^5^Research Center of Psychiatry and Behavioral Sciences, Tabriz University of Medical Sciences, Tabriz, Iran

**Keywords:** Marriage, Men, Qualitative research, Sexual behavior, Women

## Abstract

**Background:**

A decline in sexual intimacy within marital relationships can significantly impact the overall dynamics of the partnership, potentially leading to a progressive deterioration of emotional and physical closeness between couples. This study sought to examine married individuals’ perceptions of the factors contributing to diminished sexual intimacy, as well as the barriers impeding its sustenance. By elucidating these dimensions, the research aims to provide a nuanced understanding of the psychosocial and interpersonal mechanisms underlying this phenomenon.

**Methods:**

Employing a qualitative design, the study utilized a conventional content analysis approach to investigate these phenomena. A purposive sample of 26 married men and women participated in the study, with data collected through individual semi-structured interviews. Concurrent analysis was performed during data collection, with MAXQDA 2020 software used for textual data management and organization.

**Results:**

Thematic analysis yielded five principal themes (with fourteen sub-themes) characterizing factors contributing to diminished sexual intimacy. These comprised: (1) sex drive mismatch, (2) lack of perceived emotional intimacy, (3) sexual dissatisfaction, (4) restrictive sexual stereotypes, and (5) sexual nostalgia. The findings indicate that diminished sexual intimacy arises from interacting intrapersonal, interpersonal, and sociocultural factors, which cumulatively affect sexual relationship quality and, by extension, marital intimacy.

**Conclusion:**

In light of these findings, it is recommended that sexual health delivery systems prioritize implement targeted couple consultations addressing multidimensional barriers to intimacy (psychological, relational, and societal). Such evidence-based interventions could enhance sexual and marital satisfaction by providing comprehensive support tailored to couples’ needs.

## Introduction

 Marital intimacy is a cornerstone of marital stability, integrating emotional bonding, physical connection, mutual sentiment, and sexual expression, all of which contribute to the psychological and physical well-being of couples.^1^ It also helps mitigate daily stressors, enhancing marital satisfaction.^2^ Emotional and sexual intimacy demonstrate particularly strong associations with marital satisfaction.^3^ Sexual intimacy - the physical expression fostering emotional communication and mutual desire - plays a vital role in family functioning.^4^ While sexual desire refers to the motivation for sexual activity,^5,6^ sexual intimacy involves enduring relational bonds and heightened physiological sensations.^7^

 Intimacy issues often manifest as sexual problems in strained relationships. When medical causes are ruled out, intimacy-related challenges are frequently identified as key contributors to sexual difficulties.^8,9^ Diminished sexual intimacy may intensify marital conflict, precipitating psychological distress (anxiety, depression) and physiological impairments.^8,10^ Studies associate 50-60% of divorces and 40% of extramarital affairs with sexual dissatisfaction,^3^ prevalent among 70% of women.^11^

 Perceptions of sexual intimacy vary significantly by age, gender, education, sociocultural context, and religious background, lacking universal consensus.^12^ In Iran, religious and sociocultural norms substantially shape sexual attitudes.^13^ Certain sexual practices (oral/anal sex, masturbation, female circumcision) are frequently stigmatized as deviant or unethical.^14-16^ These norms, reinforced by Islamic legal restrictions, create barriers to open discussions about sexual desire and intimacy.^17,18^ In Iran, three primary discourses shape understandings of sexuality: (1) state frameworks rooted in Shi’a Islamic jurisprudence, (2) societal norms, and (3) the perspectives of the post-revolutionary generation.^19^ Shi’a clerics have reinterpreted Islamic texts to engage with modern perspectives on sexuality, blending traditional religious frameworks with contemporary knowledge.^20,21^ This dynamic interplay highlights the evolving nature of sexual discourses in Iran. However, the cultural, religious, and social context often suppresses open expression of sexual desire, hindering intimacy and increasing familial instability. Existing research on sexual intimacy in Iran has predominantly utilized medical and quantitative approaches, frequently overlooking individual lived experiences.^3,22^ Qualitative studies have focused on marital satisfaction or elderly populations,^23,24^ leaving a gap in understanding the barriers to intimacy among married couples. Given Iran’s multi-ethnic diversity, with each group having distinct cultural practices,^25^ culture-based qualitative research is essential to address these complexities.

 This study aims to explore the barriers to sexual intimacy through the perspectives of married men and women in Iran, focusing on their lived experiences and perceived challenges. By doing so, it seeks to provide insights that can inform health education policies and therapeutic interventions tailored to the Iranian context, ultimately addressing the cultural and social dynamics that influence sexual intimacy.

## Methods

###  Participants and procedures

 A qualitative study employing a conventional content analysis approach was conducted to explore married men’s and women’s perceptions of declining sexual intimacy. Given the limited research literature on barriers to sexual intimacy in Iran, this methodological approach led to new insights into this topic by capturing the unique perspectives of the participants without imposing preconceived categories or theoretical perspectives.^26^ Graneheim et al^27^ found that individual needs related to territoriality, such as identity, autonomy, privacy, and security, can constitute themes. They used an inductive approach in abstracting semantic units into codes.

 The study sample comprised purposively selected married men and women referred to a sexual health clinic in Tabriz, Iran. This clinic offers comprehensive services, including marriage counseling, couples and family therapy, and the diagnosis and treatment of sexual dysfunctions in both men and women, provided by a multidisciplinary team of medical and behavioral science specialists. To enhance sample diversity, purposive sampling with maximum variation was employed, accounting for key demographic factors such as age, gender, educational attainment, and marital duration. Inclusion criteria required participants to be sexually active, married for at least one year, and free from biological or sexual dysfunction, as well as infertility. Participants presenting with sexual dysfunctions of physiological etiology (e.g., erectile dysfunction or vaginismus) were excluded from the study. This exclusion criterion was implemented based on empirical evidence indicating that such conditions may precipitate behavioral modifications, including intimacy avoidance, which can significantly impair dyadic relationships and compromise emotional and sexual intimacy between partners.^8,9^ By excluding these cases, the study aimed to focus on non-physiological barriers to sexual intimacy, thereby providing a clearer understanding of the psychosocial and relational factors influencing this phenomenon.

###  Data collection 

 Data collection and analysis occurred concurrently between February and August 2022. Following ethical approval and institutional permissions, potential participants meeting inclusion criteria were identified through clinic visits and subsequently contacted via telephone. Semi-structured interviews were conducted by the first author, a researcher trained in qualitative methods and certified through sexology workshops. The in-depth interviews, performed individually, employed established qualitative interviewing techniques to ensure methodological rigor.

 Interviews commenced with broad, open-ended questions: *“Could you please describe your experiences regarding your sexual life? Do you feel a sense of intimacy and closeness in your sexual relationship? What factors do you believe contribute to a decline or lack of intimacy in your sexual relationship?”* Follow-up questions were customized based on responses. Probing techniques *(“Could you elaborate further? Can you provide an example of this issue? What do you mean by that?”*) facilitated deeper exploration. This semi-structured approach balanced consistency with responsiveness to individual narratives, enhancing data richness while maintaining methodological rigor.

 Interview scheduling and locations were mutually agreed upon by participants and researchers. Sessions lasted 30-70 minutes (mean = 50 minutes) in private, participant-selected settings. Audio recordings were securely stored on password-protected computers, assigned numerical codes, and transcribed verbatim. Transcripts underwent member checking (participant review/editing) before subsequent interviews. All audio files were permanently deleted post-analysis. Following qualitative methodology principles where predetermined sample sizes are inappropriate, recruitment continued until thematic saturation (no new concepts emerging) was achieved at Interview 23. Three additional confirmatory interviews were conducted (total N = 26). See Table 1 for demographic details.

**Table 1:** Demographic profile of the participants (n = 26)

**Participant code**	**sex**	**Age (year)**	**Spouse age (year)**	**marriage duration**	**Education**	**Job**	**Number of children**
1	Female	32	42	12	Bachelor	Housekeeper	1
2	Female	20	22	2	Diploma	Dentist assistant	0
3	Female	53	60	35	primary	Retired	2
4	Female	23	30	2	Bachelor	Housekeeper	0
5	Male	46	40	15	Bachelor	Self-employed	2
6	Male	30	23	3	Bachelor	Self-employed	0
7	Female	44	30	4	Bachelor	Librarian	0
8	Male	34	36	10	Bachelor	Self-employed	0
9	Female	36	34	10	Bachelor	Lawyer	0
10	Female	30	35	5	Diploma	Housekeeper	0
11	Male	31	27	2	Bachelor	Self-employed	0
12	Female	27	31	2	Bachelor	Housekeeper	0
13	Female	32	42	12	Bachelor	Housekeeper	0
14	Female	30	41	14	Diploma	Housekeeper	2
15	Male	41	30	14	Master	Employee	2
16	Female	30	35	8	Master	Writer	0
17	Female	30	42	14	Master	Hairdresser	0
18	Male	35	30	8	Master	Publisher	0
19	Male	25	25	2	Doctoral	Physician	0
20	Female	40	45	15	Master	Librarian	2
21	Female	37	41	12	Doctoral	professor	2
22	Male	26	24	6	Diploma	Self-employed	1
23	Female	25	25	2	Doctoral	physician	0
24	Male	28	23	3	Diploma	unemployment	0
25	Male	30	24	4	Bachelor	manual worker	0
26	Female	24	26	6	Diploma	Self-employed	1

###  Data analysis

 All interviews were transcribed verbatim and converted to textual data. The researcher conducted multiple close readings to comprehend participants’ perspectives. Textual data were then segmented into meaningful units and coded to capture content dimensions. After this open coding, the lists of codes were grouped based on similarities as categories, and these categories were grouped as main themes under higher-order headings. Data analysis was facilitated using MAXQDA 2020 software for systematic text management. Table 2 illustrates a representative example of this analytical process.

**Table 2:** Process of main categories

**Main categories**	**Sub-categories**	**code**	**Meaning units**
Sexual Dissatisfaction	Negative body image	Lack of sexual attraction for the husband	*“I feel my husband does not like my physique, and I am not attractive to him.”*
	Lack of sexual impulses and desires	Not satisfying a man's sexual thirst by seeing his wife	*“Seeing my wife, the stimulus that causes a pleasurable relationship does not happen to me. I cannot reach the necessary sexual arousal that prepares me for pleasurable sex, so I do not enjoy sex. ”*
	Sexually Discouraging Behaviors of Partners	Male inappropriate reaction to female sexual demands	*“I was preparing myself, putting on my make-up, and going to him for a sexual relationship, but my husband was so cold, and he would fight with me and say do not come to me without coordination. These cold behaviors have made me no longer want to have a relationship.”*
	Sexual performance anxiety	Decreased relationship pleasure due to fear of pregnancy	*“Fear of pregnancy has caused us to be dissatisfied with our sexual relationship; when sex is accompanied by fear; there is no more pleasure and intimacy.”*

###  Validity of data

 The study employed Guba and Lincoln’s criteria to assess data credibility.^28^ Credibility was established by: 1) purposive sampling of participants with relevant lived experiences and strong expressive ability; 2) prolonged engagement through multiple interviews; 3) memo-writing; 4) member checking; and 5) peer debriefing. The research team systematically verified interview data, analytical codes, categories, and interpretations through iterative review. Discrepancies were resolved through consensus-based discussions. Maximum variation sampling ensured socioeconomic diversity among participants, enhancing the study’s transferability. To enhance dependability, all authors engaged in collaborative analysis and coding, incorporating all research team members’ perspectives. Transferability was ensured through comprehensive documentation of contextual factors, participant characteristics, and observed non-verbal behaviors. Confirmation of the original themes was strengthened via: 1) member validation of emergent themes through separate participant confirmations; 2) researcher triangulation through expert panel consensus on coding; and 3) methodological triangulation combining interview guides with non-participant observation. The guidelines of consolidated criteria for reporting qualitative research (COREQ) were used for providing this manuscript.^29^ These systematic approaches enhanced the study’s trustworthiness while maintaining methodological transparency across all research phases.

###  Ethical considerations

 The study received institutional review board approval. The aims and process of the study were explained to the participants, and written informed consent was obtained. Prior to interviews, participants provided explicit permission for audio recording. To ensure confidentiality, all recordings were anonymized using unique numerical identifiers rather than personal information, with access restricted to the research team.

## Results

 The study enrolled 26 married participants (16 women [61.5%], 10 men [38.5%]; mean age = 32.3 years). Complete demographic characteristics appear in Table 1. Analysis revealed 117 initial codes, yielding eleven subthemes that coalesced into five primary themes regarding perceived barriers to sexual intimacy: (1) Sex drive mismatch, (2) Lack of perceived emotional intimacy, (3) Sexual dissatisfaction, (4) Restrictive sexual stereotypes, and (5) Sexual nostalgia. These thematic categories are examined in detail in subsequent sections.

###  Sex drive mismatch

 This concept underscores the critical role of mutual responsiveness to partners’ sexual needs in cultivating relational intimacy. Disregard for a partner’s sexual desires or desire discrepancy substantially erodes perceived intimacy. This category is further subdivided into two distinct subcategories, as described below:

####  1. Unresponsiveness to the partner’s sexual expectations

 Married participants reported divergent sexual preferences and fantasies. Perceived partner unresponsiveness to these desires correlated with decreased libido and reduced intimacy potential in spousal relationships. Most female participants stressed the importance of prolonged foreplay before intercourse, reporting this need was frequently unmet by partners. They indicated that insufficient attention to their sexual preferences decreased motivation to maintain sexual intimacy in the relationship. This perceived neglect led to decreased relational engagement and sexual interest. One of the participants mentioned their experience so:

 “*I need to hug and kiss me before sex, but he does it very hard. I would like him to praise me for my beauty and body” *(P2).

####  2. Partners’ libido mismatch

 Participants reported a perceived mismatch in libido between themselves and their partners. In long-term relationships, sexual desire naturally fluctuates due to various biopsychosocial factors influencing both individual sexual drive and relational dynamics. Key contributors include hormonal changes, child arrival, relationship challenges, aging, and stress. While these factors primarily affect individuals, their consequences manifest as desire discrepancies between partners. Sexual desire discrepancy is clinically defined as a mismatch in partners’ libido.^2^ Participants noted significant age differences - particularly when the woman is older - often exacerbate these mismatches. In such cases, couples frequently struggle to align sexual needs, resulting in relationships lacking mutual passion. One of the participants who was 14 years older than her husband said:

 “*My husband has more sexual desire than me; he is younger than me and needs to have sex every day. I do not tend to have sex every day, my husband is very hot, but I feel cold” *(P7).

###  Lack of perceived emotional intimacy

 This concept highlights the pivotal role of emotional connection in facilitating sexual intimacy between partners. It posits that emotional bonding is a prerequisite for building a fulfilling sexual relationship. Marital discord and the lack of perceived affection from a partner may result in emotional and physical detachment, thereby diminishing the frequency of intimate interactions. This category is subsequently subdivided into three distinct subcategories, as detailed below:

####  1. Spouse’s negative behavioral traits

 Behavioral traits exhibit a significant association with emotional intimacy in romantic relationships. Participants—particularly female respondents—reported that antagonistic and maladaptive behaviors, including aggression, disrespect, and verbal degradation, evoked feelings of dissatisfaction and resentment toward their partners. Such adverse emotional responses frequently resulted in the avoidance of sexual intimacy. This pattern is exemplified in the following participant accounts:

 “*My husband does not behave kindly; he is aggressive; when he insults me, I get upset, and I do not like to have a relationship with him” *(P3).

 Furthermore, a partner’s psychological characteristics, including personality traits such as introversion, may significantly influence emotional attachment and sexual attitudes. Several participants reported that dispositional factors (e.g., introversion versus extroversion) shape affective expression patterns. Deficient emotional disclosure and limited sharing of personal experiences with one’s partner were perceived to erode relational intimacy, potentially adversely affecting sexual dynamics within the relationship.

####  2. The shadow of couples’ conflicts on sexual relations 

 A harmonious relationship serves as a fundamental pillar for satisfying sexual activity within a couple. In contrast, interpersonal conflicts frequently correlate with diminished sexual relationship quality. Research participants across multiple studies have indicated that recurrent daily arguments significantly reduce sexual motivation. Even when intercourse occurs under such circumstances, it is frequently characterized as obligatory, deficient in emotional connection, sexual desire, and mutual satisfaction—often due to intrusive thoughts about recent conflicts during intimate encounters. Notably, economic strain emerged as a predominant contributor to marital discord in participant reports. Financial pressures were found to cognitively preoccupy individuals, consequently impairing emotional bonding and attenuating sexual desire. One participant highlighted this issue, stating, *“Financial concerns have taken over our minds, affecting our emotional relationship and leaving us with no desire for sexual intimacy”* (P6).

####  3. Trapped in a loveless marriage 

 Participants reported that family pressure to marry and limited freedom in choosing a spouse were important factors contributing to marriages without love. They explained that relationships formed under pressure, without true affection or emotional connection, make it difficult to develop closeness between partners. Many participants described still lacking feelings of love and affection for their spouse’s even years after marriage, which has significantly affected their sexual relationships. This is reflected in the ideas of some of the participants who expressed:

 “*From the beginning of my marital life, I did not like my husband. In my opinion, he was not acceptable. Our sexual relationship was too weak. I think it was related to my lack of love and interest in him” *(P1).

###  Sexual dissatisfaction

 Sexual dissatisfaction is characterized by persistent discontent arising from unfulfilling sexual experiences. Participants identified several contributing factors to this phenomenon, including negative body image, lack of sexual Impulses and desires, sexually discouraging behaviors of partners, and sexual performance anxiety. These factors are elaborated upon in detail below:

####  1. Negative body image

 Several participants, particularly women, reported that negative body image significantly impacted their sexual lives. They described feelings of inadequacy, perceiving their bodies as unattractive or undesirable to their partners. This self-perception frequently contributed to avoidance of sexual intimacy due to heightened insecurity and self-consciousness during intimate encounters. As one participant noted*, “I don’t feel confident about my body, and this makes it hard for me to feel close to my husband sexually.” (P 4).*

 These findings highlight the significant impact of body image perceptions on both sexual satisfaction and relationship intimacy.

####  2. Lack of sexual impulses and desires

 Multiple participants reported experiencing a marked reduction in sexual arousal relative to earlier life stages. They described a perceived loss of the passion and eagerness for sexual activity that they had previously enjoyed. This decline in sexual desire resulted in diminished satisfaction and pleasure within intimate relationships, thereby exacerbating overall sexual dissatisfaction. As one participant articulated, *“I no longer feel the same excitement or desire for intimacy as I used to. It’s hard to find pleasure in our relationship now, and it leaves me feeling unsatisfied”* (P11). These findings underscore the substantial influence of diminished sexual impulses on both interpersonal relationships and individual psychological well-being.

####  3. Sexually discouraging behaviors of partners

 Empirical findings indicate that participants frequently attributed diminished sexual desire to a lack of positive reinforcement from spouses regarding intimacy initiation attempts. Respondents described how repeated experiences of partner rejection elicited adverse emotional reactions, ultimately resulting in complete avoidance of sexual expression within the relationship. In this regard, one of the participants said:

 “*I was preparing myself, putting on my make-up, and going to him for a sexual relationship, but my husband was so cold, and he would fight with me and say do not come to me without coordination. These cold behaviors have made me no longer want to have a relationship” *(P3).

####  4. Sexual performance anxiety

 Sexual performance anxiety encompasses distress experienced during sexual activity, which may contribute to sexual dysfunction. Both male and female participants reported that such anxiety significantly reduced their sexual enjoyment, often leading to frustration and subsequent avoidance of sexual relations. Notably, concerns about unintended pregnancy emerged as a key exacerbating factor, increasing stress during intercourse and diminishing overall satisfaction with sexual intimacy. In this regard, one participant expressed:

 “*Fear of pregnancy has caused us to be dissatisfied with our sexual relationship; when sex is accompanied by fear; there is no more pleasure and intimacy” *(P18).

###  Restrictive sexual stereotypes

 This analysis underscores how culturally entrenched gender norms and religious ideologies shape societal frameworks. The construct is further operationalized through two distinct subcategories:

####  1. Guilt associated with sexual fantasizing

 Participants reported experiencing guilt associated with sexual fantasies during intercourse, which they attributed to perceptions of these thoughts as deviant, ethically inappropriate, or doctrinally forbidden. This guilt was frequently associated with reduced sexual satisfaction and impaired pleasurable experiences.

####  2. Non-expression of sexual desire by women

 Within Iran’s traditional cultural framework, women were historically positioned in a passive sexual role, while men predominantly assumed the role of initiators. Although these dynamics are evolving—with growing expectations for women to participate more actively in initiating sexual encounters—some women continue to experience shame or reluctance in voicing their sexual desires. Male participants perceived this hesitation as an impediment to sexual intimacy, with several expressing frustration regarding their partners’ difficulty in communicating sexual needs. Consequently, the responsibility for initiation remains disproportionately placed on men, contributing to relational tension.

###  Sexual nostalgia

 Sexual nostalgia denotes the reminiscence of satisfying sexual experiences occurring prior to marriage. Participants reported that the lack of comparable experiences within their marital relationships impaired their capacity to establish sexual intimacy with their spouses. Commonly cited nostalgic references included pleasure of masturbation, the pleasure of watching pornography, and pleasure of previous sexual relationships - all of which stood in contrast to their present marital sexual experiences.

####  1. Pleasure of masturbation

 Several participants indicated that solitary sexual practices during premarital periods yielded higher satisfaction than conjugal intercourse post-marriage. They characterized masturbation as an effective mechanism for attaining optimal sexual arousal - a peak physiological response they perceived as unachievable through marital sexual activity.

 “*I felt good when I masturbated; I enjoyed it, I was reaching the peak of excitement, like people who get drunk, but in sex with my wife, I do not get the sexual pleasure that I had in masturbating. I do not know what is missing; it is not exciting” *(P12).

####  2. The pleasure of watching pornography

 Multiple male participants reported that pornography consumption constituted a significant component of their sexual experiences, serving to augment both pleasure and arousal. However, this practice frequently elicited spousal disapproval, resulting in interpersonal conflict and diminished emotional and sexual intimacy within marital relationships.

####  3. Pleasure of previous sexual relationships

 Participants reported that satisfying sexual experiences in previous relationships created expectations that remained unfulfilled within their current marital context. This unmet expectation continuum was associated with diminished sexual satisfaction and impaired intimacy development between partners.

 “*Before marriage, I had sex with my boyfriend (not my current husband), which I enjoyed and led to the formation of expectations about a sexual relationship, but I do not enjoy sex with my husband because he does not meet my sexual expectations, I don’t get that pleasurable feeling in a current sexual relationship” *(P13).

## Discussion

 This study investigated the perceived barriers to sexual intimacy among married men and women in Iran, identifying five primary categories: *Sex drive mismatch*, *Lack of perceived emotional intimacy*, *Sexual dissatisfaction*, *Restrictive sexual stereotypes*, and *Sexual nostalgia*. These findings highlight the complex interplay of biological, psychological, and sociocultural factors shaping sexual intimacy within marital relationships. (1) discrepancies between desired and actual frequency of sexual behaviors (e.g., masturbation, sexual fantasies, kissing), (2) subjective reports of sexual desire, and (3) perceived differences in sexual desire.^6,30^ Misconceptions regarding gender differences may further impede couples from establishing mutually satisfying sexual relationships.^31^ For instance, biological factors such as age differences may result in the neglect of sexual preferences and desires, thereby diminishing sexual motivation and intimacy.^32^ Notably, age differences—particularly among women—play a significant role in shaping couples’ perceptions of sexual compatibility.

 Lack of emotional intimacy was identified as a significant barrier to sexual intimacy in marital relationships. Marital conflicts—including aggressive behaviors, financial stressors, and persistent intrusive thoughts—were found to diminish sexual desire and impair sexual satisfaction.^33,34^ Gender differences were evident in responses to relational aggression: men tended to associate withdrawal with reduced physical intimacy, whereas women perceived it primarily as emotional disengagement, both ultimately leading to decreased sexual activity.^35^ Exposure to relational violence was particularly detrimental, as it redirected focus toward self-protection during sexual encounters rather than emotional bonding.^36^ Furthermore, financial strain emerged as a critical exacerbating factor, increasing hostility and further disrupting both emotional and sexual intimacy within couples. Empirical evidence demonstrates that economic adversity significantly reduces orgasm frequency and sexual satisfaction, with men being particularly affected due to difficulties in emotional expression during financial strain.^37^ Research further establishes a strong association between increased physical/verbal aggressions and reduced marital and sexual satisfaction.^38,39^

 The persistence of patriarchal ideologies exacerbates marital conflict, particularly through gender-based violence. These attitudes, rooted in gender norms that subordinate women and empower men. Perpetuate intimate terrorism—violent behaviors employed to maintain patriarchal dominance.^40^ Within the Iranian context, the intersection of religious doctrine and patriarchal social frameworks amplifies gender inequities, a finding unanimously reported by all 26 study participants (16 female, 10 male).

 Negative body image emerged as a significant barrier to sexual intimacy, with particularly pronounced effects among female participants. Women’s persistent self-evaluation of physical appearance during sexual activity frequently leads to sexual self-objectification, subsequently diminishing both sexual desire and satisfaction.^41,42^ This cognitive preoccupation creates distraction and performance anxiety, interfering with sexual enjoyment.^43^ Additionally, male partners’ sexually discouraging behaviors, often rooted in cultural norms, can evoke feelings of rejection and diminish sexual desire among women.^44^ Within the Iranian context, patriarchal norms actively constrain women’s sexual expression, with such assertiveness often met with negative spousal responses.^45^ Compounding these issues, the absence of sexual synchrony—encompassing situational, behavioral, and attitudinal dimensions—intensifies relational dissatisfaction. Repeated rejection experiences ultimately create a cycle of sexual avoidance.^46^ These patterns were reflected in reports from 21 participants (15 women, 6 men).

 In Iran, sexuality is heavily influenced by restrictive sexual stereotypes rooted in culture, religious doctrine, educational systems, and familial structures.^13,33^ Negative religious attitudes toward sexual fantasies often induce guilt, as such thoughts are culturally stigmatized as immoral.^47^ Iran’s Islamic theocracy shapes sexual policies through religious-national narratives, reinforcing passive sexual socialization.^48^ Traditional gender roles prescribe markedly different sexual scripts: women are socialized into passive receptivity, while men are culturally sanctioned as exclusive initiators.^49^ This dichotomy persists despite research indicating male preferences for mutual sexual expression,^50^ Patriarchal structures create significant barriers for women attempting to articulate both erotic and non-erotic emotions, resulting in constrained sexual agency.

 Study participants commonly reported sexual nostalgia, indicating dissatisfaction stemming from limited sexual variety in their current relationships.^51^ Notably, pornography consumption - predominantly among male participants - fostered unrealistic expectations regarding physical appearance and intimate behaviors, adversely affecting authentic sexual connections.^52^ Within Iran’s religious-cultural context, such practices carry significant stigma, frequently precipitating interpersonal discomfort and marital discord.^45^ This sexual repression systematically erodes relational intimacy.

 The analysis further revealed how structural gender inequities impair sexual fulfillment. Heteronormative socialization compels women to adopt reactive sexual roles to avoid social censure, contributing to reduced orgasmic frequency and sexual dissatisfaction.^5,53^ Many women consequently view sexual activity as spousal duty rather than mutual pleasure - a perspective reinforced by threats of domestic violence and constrained societal agency.^54^ These normative frameworks disproportionately burden women with relationship maintenance responsibilities.^55^

 This investigation’s primary strength resides in its qualitative methodology, which facilitated nuanced examination of the culturally sensitive subject of marital sexual intimacy in Iran. However, several methodological constraints warrant consideration. The use of purposive sampling restricted participation to heterosexual married individuals from specific ethnic demographics, potentially limiting the findings’ generalizability. Furthermore, prevailing cultural taboos regarding sexual discourse may have resulted in underreporting of sensitive experiences. Furthermore, the exclusion of the LGBTIQA + (lesbian, gay, bisexual, transgender, intersex, queer/questioning and asexual) community due to legal and religious constraints, restricts the study’s inclusivity. Despite these limitations, the study offers valuable insights into the barriers to sexual intimacy in Iranian marital relationships, highlighting the need for culturally adapted interventions to manage these challenges.

## Conclusion

 Marital sexual intimacy frequently declines over time, representing a substantial concern for families and mental health professionals as it contributes to marital discord and serves as a predictor of divorce. This investigation offers crucial insights into obstacles to sexual intimacy among Iranian married couples, where traditional religious norms regulate sexuality through cultural prohibitions, restrictive policies, and spiritual perspectives, thereby compounding the complexity of the issue. Addressing these challenges necessitates a systematic approach involving: 1) examination of social structures and cultural beliefs within Muslim communities to identify root causes, and 2) development of strategic frameworks for comprehensive sexual education programs.

 The findings provide important perspectives on intimacy barriers, assisting families, policymakers, and clinicians in formulating innovative solutions. Furthermore, they inform the development of culturally appropriate interventions through the integration of indigenous values into therapeutic approaches. This dual-focused methodology ensures interventions are both clinically effective and culturally congruent, ultimately promoting healthier sexual relationships and more resilient family systems within Iran’s sociocultural context.

## Competing Interests

 Prof. Hamid Allahverdipour is the Editor-in-Chief in *Health Promotion Perspectives*. The authors report there are no competing interests to declare.

## Data Availability Statement

 All data on which this article is based are included within the article.

## Ethical Approval

 The ethics committee of Tabriz University of Medical Sciences (TBZMED) approved the study protocol (Ethics Code: IR.TBZMED.REC.1398.558). The aims and process of the study were explained to the participants, verbal and written informed consent was obtained. Before interviews, participants received permission to use a voice recorder to record the discussions. To protect privacy, all interviews were recorded anonymously using code numbers.

## References

[1] Kamali Z, Allahyar N, Ostovar S, Alhabshi SM, Griffiths MD (2020). Factors that influence marital intimacy: a qualitative analysis of Iranian married couples. Cogent Psychol.

[2] Kardan-Souraki M, Hamzehgardeshi Z, Asadpour I, Mohammadpour RA, Khani S (2016). A review of marital intimacy-enhancing interventions among married individuals. Glob J Health Sci.

[3] Salehi Moghaddam F, Torkzahrani S, Moslemi A, Azin SA, Ozgoli G, Joulaee Rad N (2020). Effectiveness of sexual skills training program on promoting sexual intimacy and satisfaction in women in Tehran (Iran): a randomized clinical trial study. Urol J.

[4] Theiss JA, Nagy ME (2010). Actor-partner effects in the associations between relationship characteristics and reactions to marital sexual intimacy. J Soc Pers Relat.

[5] van Anders SM, Herbenick D, Brotto LA, Harris EA, Chadwick SB (2022). The heteronormativity theory of low sexual desire in women partnered with men. Arch Sex Behav.

[6] Mark KP (2015). Sexual Desire Discrepancy. Curr Sex Health Rep.

[7] Ferreira LC, Narciso I, Novo RF, Pereira CR (2014). Predicting couple satisfaction: the role of differentiation of self, sexual desire and intimacy in heterosexual individuals. Sexual and Relationship Therapy.

[8] Bagarozzi DA. Enhancing Intimacy in Marriage: A Clinician’s Guide. Routledge; 2014.

[9] Gholamrezaei S, Hosseini H, Kariminejad K (2017). The impact of cognitive-behavioral training program on marital satisfaction of women with hypoactive sexual desire. Family Pathology, Counselling & Enrichment Journal.

[10] Villegas R. The Relationship Between Quality of Paternal Relationship and Paternal Physical Proximity and Women’s Romantic Attachments and Sexuality [dissertation]. San Francisco Bay: Alliant International University; 2006.

[11] Abdoly M, Pourmousavi L (2013). The relationship between sexual satisfaction and education levels in women. Int J Womens Health Reprod Sci.

[12] Bowling J, Blekfeld-Sztraky D, Simmons M, Dodge B, Sundarraman V, Lakshmi B (2020). Definitions of sex and intimacy among gender and sexual minoritised groups in urban India. Cult Health Sex.

[13] Abdolmanafi A, Nobre P, Winter S, Tilley PJ, Ghorban-Jahromi R (2018). Culture and sexuality: cognitive-emotional determinants of sexual dissatisfaction among Iranian and New Zealand women. J Sex Med.

[14] Maasoumi R, Taket A, Zarei F (2018). How Iranian women conceptualize the role of cultural norms in their sexual lives. Sex Cult.

[15] Motamedi M, Merghati-Khoei E, Shahbazi M, Rahimi-Naghani S, Salehi M, Karimi M (2016). Paradoxical attitudes toward premarital dating and sexual encounters in Tehran, Iran: a cross-sectional study. Reprod Health.

[16] Shaygani F, Zahedroozegar MH, Honarvar B, Shaygani MR (2021). Eradicating female genital mutilation in Iran: actions to be done. Arch Iran Med.

[17] Arjmand R, Ziari M (2018). Sexuality and concealment among Iranian young women. Sexualities.

[18] Javadivala Z, Allahverdipour H, Asghari Jafarabadi M, Azimi S, Gilani N, Chattu VK (2021). Improved couple satisfaction and communication with marriage and relationship programs: are there gender differences? A systematic review and meta-analysis. Syst Rev.

[19] Saeidzadeh Z (2016). Transsexuality in contemporary Iran: legal and social misrecognition. Fem Leg Stud.

[20] Mohammadi E, Durnova A (2021). Policy expertise and culture: the case of “civil sexuality” in Iran. Int Rev Public Policy.

[21] Merghati Khoei E, Moeini B, Barati M, Soltanian AR, Shahpiri E, Ghaleiha A (2019). A qualitative inquiry of sexuality in Iranian couples using the information-motivation-behavioral skills paradigm. J Egypt Public Health Assoc.

[22] Azimi S, Javadivala Z, Alizadeh Mizani M, Pourrazavi S, Fathifar Z, Chattu VK, et al. A glance at the efficiency of marital sexual intimacy interventions: the different outcomes on couples and women. Fam J. 2023:10664807231174220. doi: 10.1177/10664807231174220.

[23] Garrett D (2014). Psychosocial barriers to sexual intimacy for older people. Br J Nurs.

[24] Namvaran Gerami K, Moradi A, Farzad V, Zahrakar K (2018). The identification of the dimensions of Iranian couples’ marital intimacy. The Women and Family Cultural Education.

[25] Majbouri M, Fesharaki S (2019). Iran’s multi-ethnic mosaic: a 23-year perspective. Soc Indic Res.

[26] Hsieh HF, Shannon SE (2005). Three approaches to qualitative content analysis. Qual Health Res.

[27] Graneheim UH, Lindgren BM, Lundman B (2017). Methodological challenges in qualitative content analysis: a discussion paper. Nurse Educ Today.

[28] Guba EG (1981). Criteria for assessing the trustworthiness of naturalistic inquiries. Educ Technol Res Dev.

[29] Tong A, Sainsbury P, Craig J (2007). Consolidated criteria for reporting qualitative research (COREQ): a 32-item checklist for interviews and focus groups. Int J Qual Health Care.

[30] Bridges SK, Horne SG (2007). Sexual satisfaction and desire discrepancy in same sex women’s relationships. J Sex Marital Ther.

[31] Miller SA, Byers ES (2004). Actual and desired duration of foreplay and intercourse: discordance and misperceptions within heterosexual couples. J Sex Res.

[32] Ghorashi Z, Merghati Khoei E (2017). Exploring the reducing satisfactory response in married women of reproductive age: qualitative study. Iran J Psychiatry Clin Psychol.

[33] Hashemiparast M, Naderi B, Chattu VK, Allahverdipour H (2024). Perceived barriers of expression of sexual desires among older adults: a qualitative study. Sexual and Relationship Therapy.

[34] Coker AL (2007). Does physical intimate partner violence affect sexual health? A systematic review. Trauma Violence Abuse.

[35] Carroll JS, Nelson DA, Yorgason JB, Harper JM, Ashton RH, Jensen AC (2010). Relational aggression in marriage. Aggress Behav.

[36] Metz ME, Epstein N (2002). Assessing the role of relationship conflict in sexual dysfunction. J Sex Marital Ther.

[37] Katz J, Carino A, Hilton A (2002). Perceived verbal conflict behaviors associated with physical aggression and sexual coercion in dating relationships: a gender-sensitive analysis. Violence Vict.

[38] Dobson K, Zhu J, Balzarini RN, Campbell L (2020). Responses to sexual advances and satisfaction in romantic relationships: is yes good and no bad?. Soc Psychol Personal Sci.

[39] Dobson K, Ogolsky B (2021). The role of social context in the association between leisure activities and romantic relationship quality. J Soc Pers Relat.

[40] Bates EA (2016). Current controversies within intimate partner violence: overlooking bidirectional violence. J Fam Violence.

[41] Ashkinazi M, Wagner SA, Cunningham K, Mattson RE (2024). Body image satisfaction and body-related partner commentary link to marital quality through sexual frequency and satisfaction: a path model. Couple Family Psychol.

[42] Mojtabayi M, Saberi H, Alizadeh A (2015). The role of sexual–self schema and body image on women sexual function. J Health Psychol.

[43] Cherkasskaya E, Rosario M (2017). A model of female sexual desire: internalized working models of parent-child relationships and sexual body self-representations. Arch Sex Behav.

[44] Barzoki MH, Seyedroghani N, Azadarmaki T (2013). Sexual dissatisfaction in a sample of married Iranian women. Sex Cult.

[45] Rahbari L. Sexuality in Iran. In: The Wiley Blackwell Encyclopedia of Family Studies. Wiley; 2016. p. 1769-72.

[46] Gillespie BJ (2017). Sexual synchronicity and communication among partnered older adults. J Sex Marital Ther.

[47] Cado S, Leitenberg H (1990). Guilt reactions to sexual fantasies during intercourse. Arch Sex Behav.

[48] Refaie Shirpak K, Chinichian M, Maticka-Tyndale E, Eftekhar Ardebili H, Pourreza A, Ramenzankhani A (2008). A qualitative assessment of the sex education needs of married Iranian women. Sex Cult.

[49] Maasoumi R, Lamyian M, Khalajabadi Farahani F, Montazeri A (2013). Women’s perception of sexual socialization in Iran: a qualitative study. J Qual Res Health Sci.

[50] Ziegler A, Conley TD. The importance and meaning of sexual fantasies in intimate relationships. In: Aumer K, ed. The Psychology of Love and Hate in Intimate Relationships. Cham: Springer International Publishing; 2016. p. 29-45. doi: 10.1007/978-3-319-39277-6_3.

[51] Muise A, Kim JJ, Debrot A, Impett EA, MacDonald G (2020). Sexual nostalgia as a response to unmet sexual and relational needs: the role of attachment avoidance. Pers Soc Psychol Bull.

[52] Wright PJ (2013). US males and pornography, 1973-2010: consumption, predictors, correlates. J Sex Res.

[53] Drolet CE, Drolet AM (2019). Self-objectification, system justifying beliefs, and the rise of labiaplasty. Soc Just Res.

[54] Amaro H, Raj A (2000). On the margin: power and women’s HIV risk reduction strategies. Sex Roles.

[55] Gill R (2009). Mediated intimacy and postfeminism: a discourse analytic examination of sex and relationships advice in a women’s magazine. Discourse Commun.

